# The Ninth International Medical Congress

**Published:** 1885-10

**Authors:** 


### EDITORIAL.

### THE NINTH INTERNATIONAL MEDICAL CONGRESS.

In another part of this number of THE JOURNAL AND EXAMINER will be found the report of the meeting, in New York, on September 3d and 4th, of the enlarged Committee of Arrangements appointed by the American Medical Association on preliminary organization of the ninth International Medical Congress. Examination of that report will make manifest the fact that that Committee acted with wisdom, and a commendable desire to rectify, as far as it was practicable to do so, mistakes that, it was apparent, had been made.

It eliminated the so-called code question; it abandoned the idea of making the congress a representative body, thus restricting membership as had never been done before, and it made the conditions of membership as liberal as in any previous congress; it declared against assigning to any one individual multiple offices, and proceeded to fill vacancies found to exist.

After it had established the sections and appointed presidents for them, it framed rules for the government of the congress, and then organized an Executive Committee, to consist of the officers of the congress, including the presidents of the sections, and authorized that Executive Committee to add to its membership a limited number of representative men in the medical profession, whose coöperation might be desired.

Having thus arranged the preliminary organization of the congress, the Committee of Arrangements considered that it had practically accomplished the work for which it had been created, and it resigned the remaining details to the Executive Committee.

This result indicates that this Committee of Arrangements, from which many seemed to fear so much, showed that it comprehended the responsibility of the important duties assigned it, and realized the difficulties under which these duties had to be performed, and that it discharged them, under these trying circumstances, in a manner that must commend itself to the thoughtful and the unprejudiced, even if they had wished to see a result different in some respects.

When it had realized the mistakes previously made, and had done what could be done to remedy them, and had faithfully discharged the trust confided to it, it surrendered its delegated powers to its successor, without having appropriated to itself the offices of the congress, which its opponents charged was one of its objects. In its comprehension of its duties, as trusted medical men, assigned a difficult task and surrounded by embarrassments, it showed capability, fidelity and a degree of unselfishness not unworthy of imitation. The result is before the medical profession of the world, and it will be judged of by its merits.

Sufficient time has now elapsed since the last annual meeting, in New Orleans, of the American Medical Association, to have given an opportunity to weigh calmly our duty as medical men. Whatever may have been the mistakes made, as many believe, at that meeting, there seems now to be no good reason why those who felt aggrieved by, or who could not approve of, some of the acts, or the results of the acts, of the Association in that meeting, should not now take part in the effort that is being made to redeem the pledges given at Copenhagen in behalf of the medical profession of the United States. The removal of the congress from the United States, as advised by some and apparently hoped for by others, would be a lasting disgrace to American physicians; and it is difficult to comprehend how any of those, whom we all respect, can so complacently anticipate such a result of the unfortunate dissension, which has already lasted too long. Americans have usually been credited with not being wanting in a desire to preserve their good name, or to keep their faith when pledged. We not only have patriotism enough but also regard enough for the honor of the medical profession of our country to be unwilling to believe that differences of opinion will be allowed to sway us to such an extent as to destroy all prospect of holding a successful congress in Washington in 1887. Let it not be said of our profession that personal pique influenced our action beyond all reason. For the good of the cause, for the sake of humanity and science and patriotism, as well as for the love of our profession and its good name, let dissension cease, and, in a spirit of justice, fairness, and conciliation, let the mistakes of the past become a matter of the past, and let a united effort be made to make the congress what it ought to be. The occasion is too great and the differences are too small to warrant jeopardizing of the prospects of the congress.

